# The antimicrobial effects of PLGA microspheres containing the antimicrobial peptide OP-145 on clinically isolated pathogens in bone infections

**DOI:** 10.1038/s41598-022-18690-y

**Published:** 2022-08-25

**Authors:** Ye Cheng, Jianhua Qin, Yuliang Huang, Tianyu Wang

**Affiliations:** 1 Department of Orthopaedics, Xinhua Hospital of Zhejiang Province, The Second Affiliated Hospital of Zhejiang Chinese Medical University, No. 318 Chaowang Road, Gongshu District, Hangzhou, 310003 Zhejiang China; 2 Hangzhou Plastic Surgery Hospital, Hangzhou, China; 3 Department of Rehabilitation, Xinhua Hospital of Zhejiang Province, The Second Affiliated Hospital of Zhejiang Chinese Medical University, Hangzhou, China No. 318 Chaowang Road, Gongshu District, 310003

**Keywords:** Drug discovery, Microbiology

## Abstract

Infection after fracture is a significant problem for the healing of fractures. Antimicrobial peptides combined with PLGA (poly-lactic-co-glycolic acid) microspheres can open new horizons for treating bone infections. Twenty rats in the control group were treated with physiologic saline solution after surgery, and 20 rats in the treatment group were treated with OP-145 PLGA microspheres and vancomycin after surgery. The biofilms from treatment and control groups were analyzed by fluorescence microscopy. Blood samples were collected at 12, 24, 36, 48, and 72 h. OP-145 PLGA microspheres showed significant inhibitory effects on clinically isolated strains (*P* < 0.05) and there were significant differences in serum CRP (*P* < 0.05) levels compared with control group. In conclusion, OP-145 PLGA microspheres could slowly release antimicrobial peptides and significantly reduce biofilm formation and levels of inflammatory factors.

## Introduction

Proteomics can help determine the relationship between protein structure and function. In recent years, more and more polypeptide and protein substances have been used in the diagnosis and treatment, or as vaccines to prevent various diseases^1^. Compared with small-molecule drugs, polypeptide and protein molecular drugs are easily degraded enzymatically, with a short biological half-life^2^. In addition, their poor diffusion and distribution coefficient make it difficult for them to penetrate through biological barriers and lipid membranes. Therefore, the question is how these biological materials can be effectively delivered to the corresponding target points, making their preparation a research hotspot.

At present, most biological drugs are used by injection in the clinic, necessitating frequent dosing and leading to patients’ poor compliance^3^. Small-molecule drugs combined with the biodegradable microsphere systems can effectively prevent the drug’s quick degradation in the body and help target effective parts in the body, with long-term, slow release of drugs^4^. AMPs could be encapsulated in a degradable sustained release carrier with the advantages of lower mass density, greater surface area and drug release kinetics^5,6^. OP-145 is a synthetic antimicrobial peptide developed from the human cathelicidin LL-37. OP-145 was more effective than LL-37 in eradicating S. aureus in wound infection models in vitro^7^. Existing peptide and protein microspheres mainly include injection of sustained-release preparations and oral and nasal inhalers^8^. Infection after fracture is a significant problem for the healing of fractures^9^. Antimicrobial peptides combined with PLGA (poly-lactic-co-glycolic acid) microspheres can open new horizons for treating bone infections^10^.

This study aimed to study the antibacterial effect of polypeptide OP-145 microspheres on clinically isolated pathogens in vivo and determine their cytotoxicity. Therefore, we determined bacterial counts in the biofilm and used confocal laser scanning microscopy (CLSM) to verify the effects of polypeptide-protein PLGA microspheres on clinically isolated pathogens. Meanwhile, cytotoxicity on osteoblasts was tested to verify the clinical value of polypeptide-protein PLGA microspheres.

## Material and methods

### Strains isolation and culture conditions

One patient with clinical infection after fracture at the Xinhua Hospital was selected to isolate the clinical pathogenic bacteria. Bacterial specimens were obtained after washing the wound’s surface vigorously by saline solution, followed by debridement of superficial exudates. The microbiological culture was carried out under microaerophilic conditions for seven days. The bacterial specimens were tested by pyrosequencing analysis of bacterial diversity, and antimicrobial susceptibility tests were tested by the disk diffusion method^11^.

### Peptide

OP-145 (acetyl-IGKEFKRIVERIKRFLRELVRPLR-amide) was synthesized by Shanghai Apeptide Co. Ltd. (Shanghai, China). OP-145 was purified by high performance liquid chromatography, and the identity was verified by SDS-PAGE. In addition, the purity of OP-145 (> 95%) and mass were confirmed by electrospray ionization mass spectrometry.

### Preparation of PLGA microspheres

OP-145 containing PLGA microspheres was prepared by a previously described methods^12,13^. Briefly, 200 mg of PLGA was dissolved in methylene chloride (45%, w/v). The OP-145 solution (0.2 mg/ml) was then added to the polymer solution to form the organic phase and mixed via vortexing. The organic phase containing both the polymer and OP-145 was then added at once to 100 mL of 0.35% (w/v) PVA solution (0.22-μm membrane filtered) to form an oil-in-water (o/w) emulsion using a homogenizer set at 14,000 rpm for 1 min. Next, leuprolide acetate microspheres with large particle sizes were prepared using a lower emulsification/size reduction force (9000 rpm for 1 min). The resultant o/w emulsion was stirred at 400 rpm for 3 h under vacuum at room temperature to allow microsphere solidification and solvent evaporation. The resultant microspheres were collected by centrifugation, washed using distilled water, and freeze-dried using a vacuum manifold. The drug release of OP-145 from PLGA microspheres was calculated by the methods of Garner J^14^. To investigate the morphology of the PLGA microparticles, the PLGA microparticles were first treated by freeze-drying, observed under a scanning electron microscope (SEM, NNS-450, FEI, USA)^15^, and analyzed by x-ray photoelectron spectroscopy (XPS)^16^.

### Animals

Sixty adult female Sprague‐Dawley (SD) rats (weighing 260–320 g) were used in this study. Twenty rats in the blank control group were treated with physiologic saline solution after surgery^17^, 20 rats in the treatment group were treated with OP-145 PLGA microspheres and 20 rats were treated with vancomycin as positive group after surgery. All surgery was performed under sodium pentobarbital anesthesia, and euthanasia was accomplished with CO_2_.

### Model validation

The rats were anesthetized via inhalation of 2% isoflurane. The surgical procedure was as described in the previous work with few modifications^18^. The skin was incised at the proximal end of the specimen to facilitate loading directly and longitudinally over the lateral upper forelimb. Dissection continued up to the fascia of the biceps and brachialis, which were then retracted to reveal the midshaft humerus. A femoral fracture was created with an electric drill, and normal saline was not ejected after the drill stopped.

An inoculum of clinically isolated bacteria (1 × 10^5^ CFU/mL) in 2 mL of normal saline solution was pipetted into the femur space to create infection models^19^. In the blank group, the fracture site were covered with phosphate buffered saline. In the other 2 groups, the fracture site were filled with OP-145 containing PLGA microspheres, or vancomycin. The surgical site was closed with Dexon 5–0 sutures. The weights of the rats were recorded at 0, 1, 2, 3, 7, and 14 d. C-reactive protein (CRP) levels were measured by enzyme-linked immunosorbent assay (ELISA) at 12, 24, 36, 48, and 72 h^20^.

### Detection of biofilm formation

The biofilms were harvested from euthanized rats on days 1, 2, and 3 to test biofilm formation in the present rat model. The specimens were prepared according to the previous methods^21^. The specimens were fixed in 2.5% glutaraldehyde at 4 °C for 1 h and stained with LIVE/DEAD staining^22^. The biofilms were observed under a Nikon 80i microscope equipped with an argon laser with an excitation wavelength of 488 nm (green fluorescence). All the images were captured and saved using Nis-Elements AR software (Nikon, Tokyo, Japan). The integrated optical density (IOD) of biofilm was evaluated using the Image-Pro-Plus^23^.

### MTT assay

The cytotoxicity of OP-145 (0.5 and 1 µg/mL) to bone marrow stromal cells (BMSCs) was assessed using an MTT cell proliferation kit (Roche Applied Science). The BMSCs were incubated at 37 °C under 5% CO_2_ for 1 and 2 days after cell inoculation as described previously. Next, a 50-mL volume of MTT working solution was added to each well, and the mixture was incubated for another 4 h. Purple crystal formazan was observed around cells at × 40 magnification under a microscope. The cell medium was carefully removed, and then 100 mL of dimethyl sulfoxide was added to each well to dissolve formazan. After 15 min of incubation at 37 °C to completely dissolve formazan, the absorbance was measured at 490 nm on an enzyme-linked immunosorbent assay (ELISA) plate reader, and the results were expressed as optical density (OD) values. The OD values were calculated from concentration–response curves and used as a measure of cellular sensitivity.

### Statistical analysis

All data are presented as a mean ± _standard deviation and most of the statistical comparisons have been made using the Manne-Whitney Rank Sum test. The data were analyzed using GraphPad Prism 5.0. One-way ANOVA and Tukey multiple comparison tests were used to compare different treatments. A P-value < 0.05 was considered statistically significant.

### Ethics approval and consent to participate

The current study was performed by approval of the Ethics Committee of the Xinhua Hospital of Zhejiang Province with approval number 20210123–12. In addition, the committee approved the utilization of human samples within this study. The clinical samples were taken from one patient with informed consent was applied from the participant. In this study, all animal care and use protocols were performed in accordance with the Regulations for the Administration of Affairs Concerning Experimental Animals approved by the State Council of People’s Republic of China. All animal experiments in this study were approved by the Animal Research Ethics Committee of Xinhua Hospital of Zhejiang Province (No. 20212123–45). And the methods were performed in accordance with the relevant guidelines and regulations. All experimental protocols were approved by the Ethics Committee of the Xinhua Hospital of Zhejiang Province. All methods are reported in accordance with ARRIVE guidelines for the reporting of animal experiments and informed consent was obtained from all subjects.

## Results

### Clinically isolated strains and antimicrobial susceptibility test

**Figure 1:**
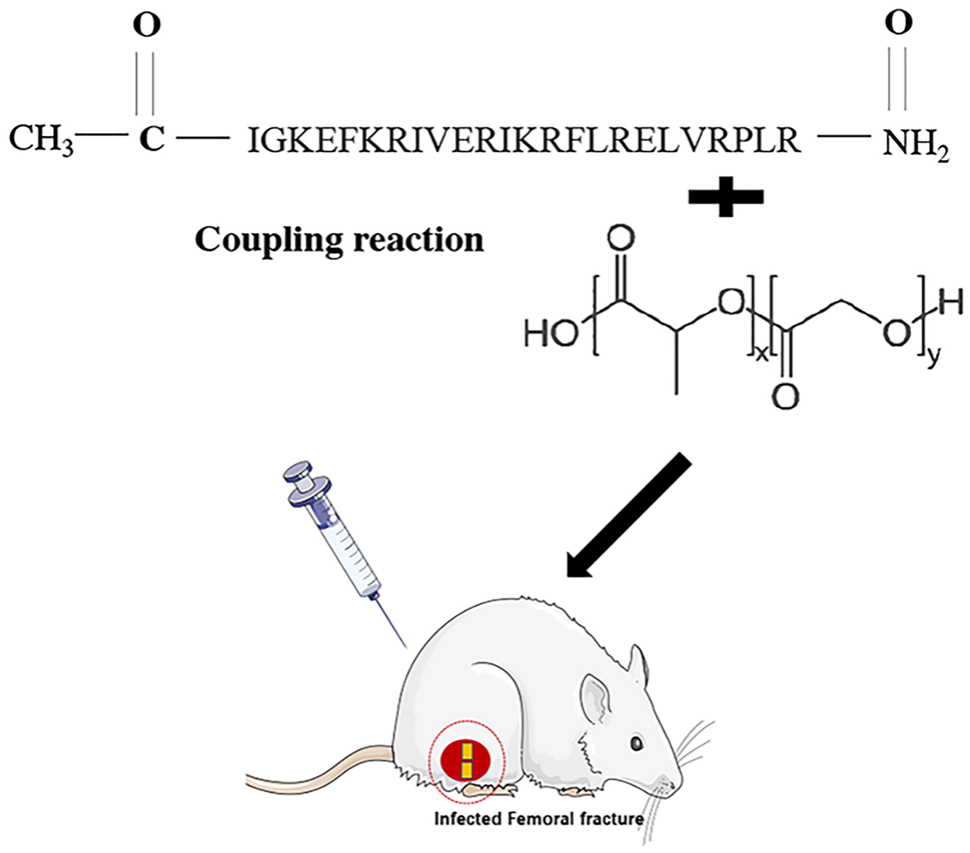
OP-145 was combined with PLGA through the amine coupling reaction for on-demand infected wound healing.

**Figure 2:**
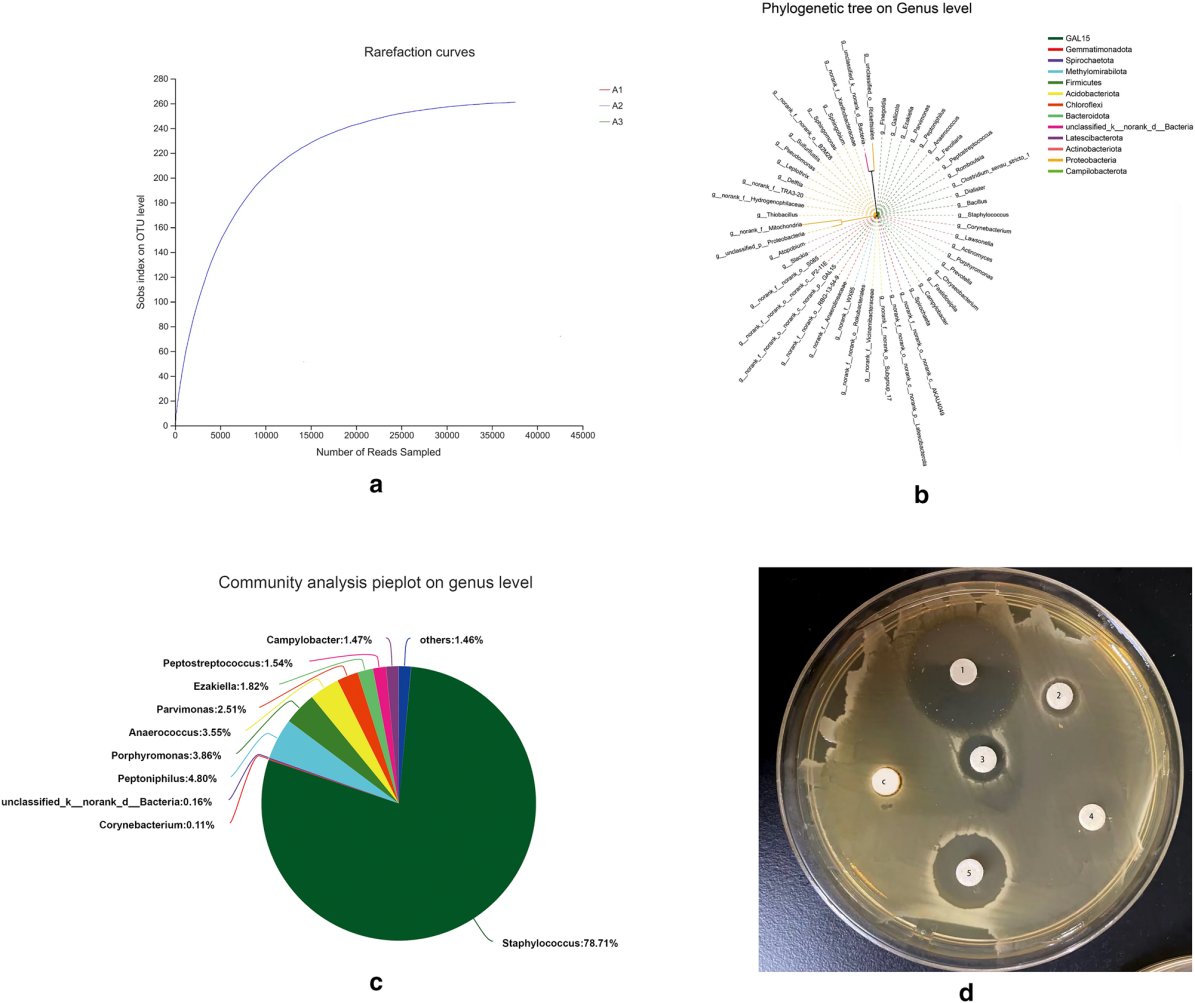
(**a**) The number of rarefaction curves in clinical isolates; (**b**) phylogenetic tree at the gene level; (**c**) the bacterial content in clinical isolates; (**d**) the KB paper method for common antibiotics sensitivity test. (1: vancomycin; 2: cefixime; 3: polymyxin; 4: tetracyclines; 5: OP-145; C: blank control).

The progress of the experiment was shown in Fig. 1. Figure 2 shows the microbial diversity of the isolated strains from patients. *S. aureus* was the main pathogen of the infection (78.71%). *Peptoniphilus* and *Porphyromonas* were the other main bacteria in the isolated strains. In the antimicrobial susceptibility test, vancomycin, OP-145, and cefixime exhibited strong antimicrobial activity on clinically isolated strains; therefore, we used vancomycin as a positive control in the following tests according to the previous study^24^.

### Preparation of OP-145 containing PLGA microspheres

**Figure 3:**
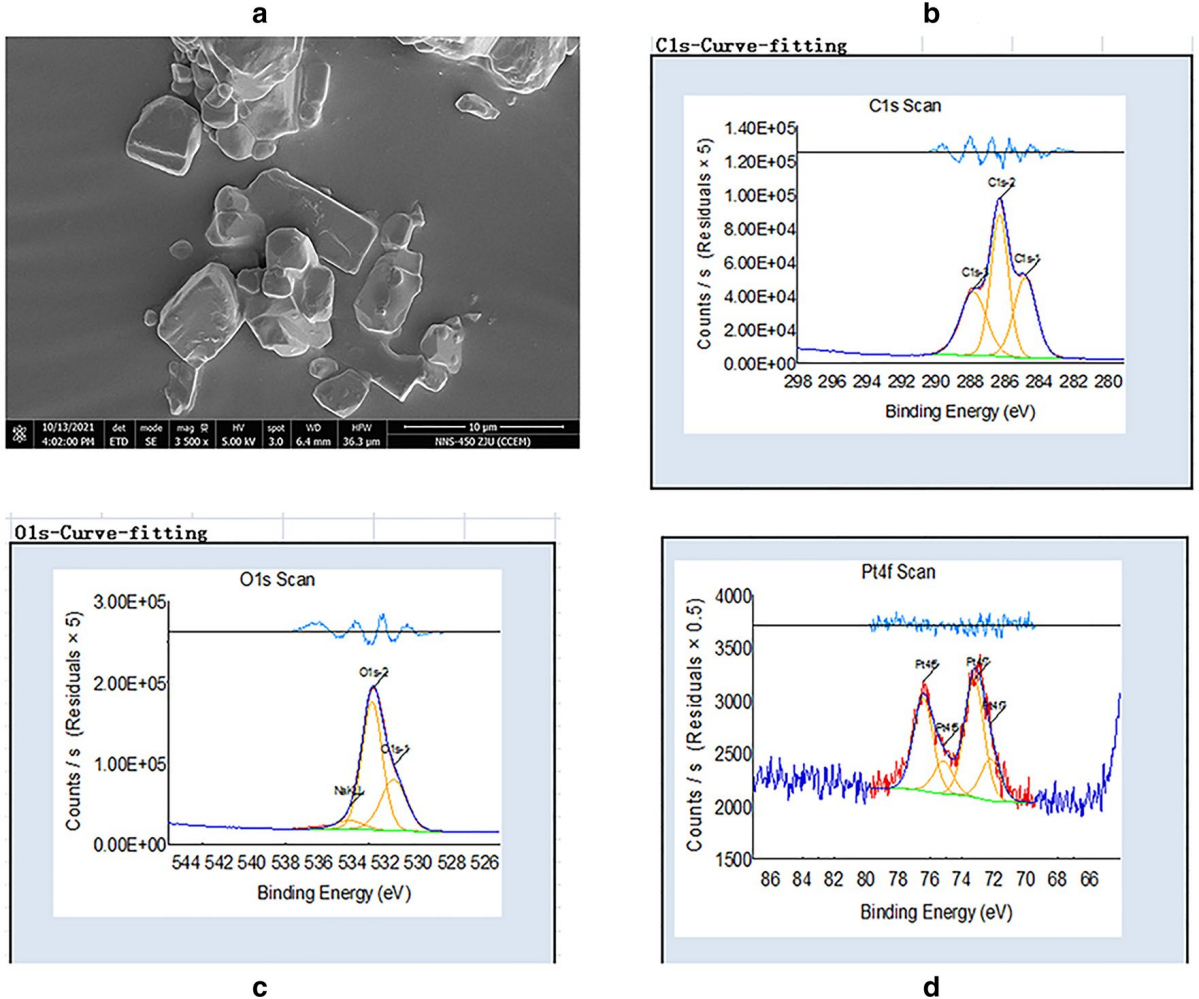
(**a**) SEM images of OP-145 PLGA; (**b**-**d**) X-ray photoelectron spectroscopy analyses of OP-145 PLGA.

The PLGA microspheres were formed through a water-in-oil method and further cross-linked and freeze-dried to obtain a porous PLGA microsphere. Under the scanning electron microscope observation, the PLGA microspheres produced by coflow shearing of coaxial nozzles exhibited well dispersed, uniform sizes and well-maintained integrity in shapes (Fig. 3a). The XPS analysis of the carbon peak (C 286 eV) and oxygen peak (O 532.0 eV) showed that the PLGA microsphere contained C and O (Fig. 3b–d). Drug.

### Evaluation of in vivo anti-biofilm activity

**Figure 4:**
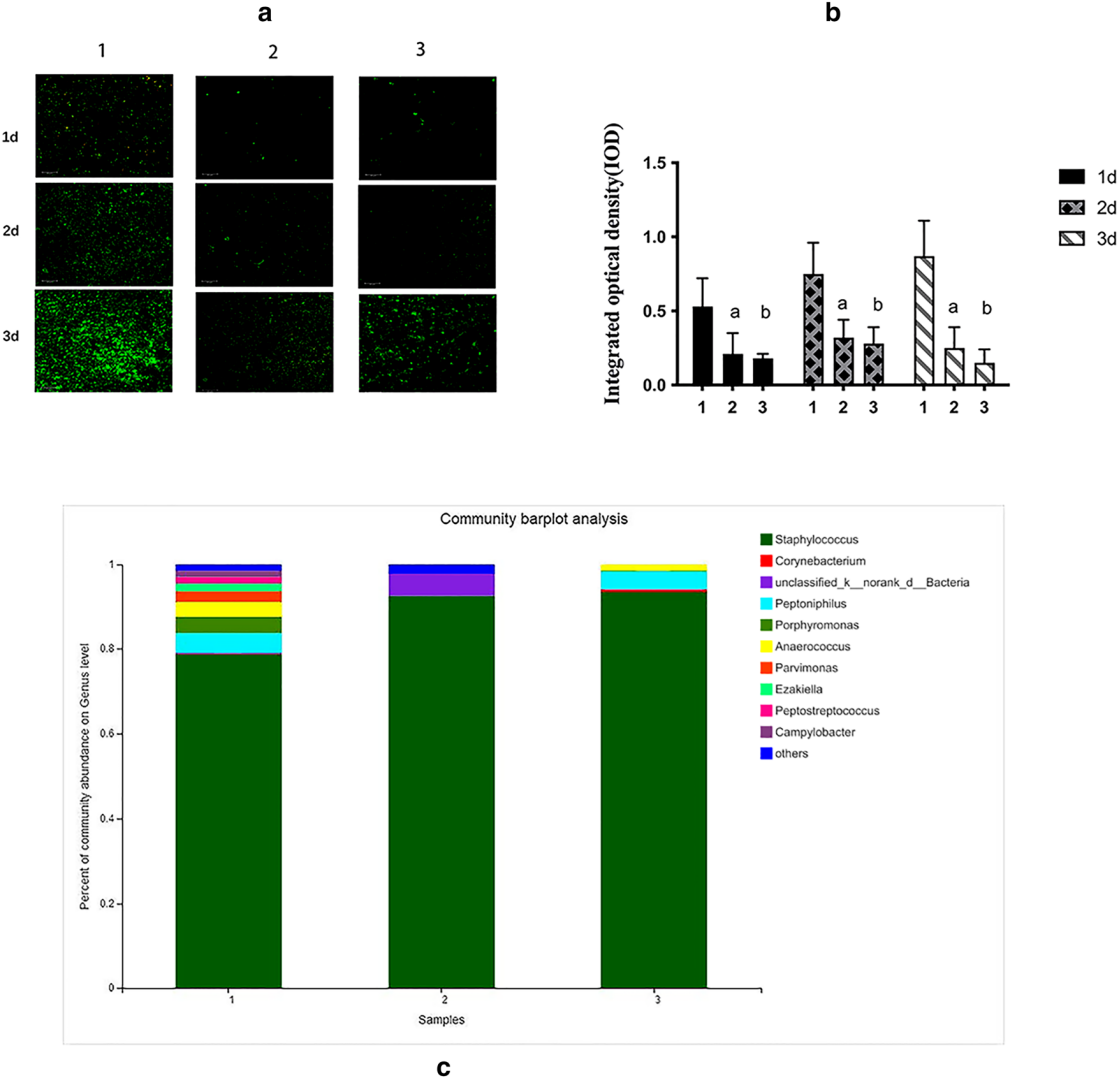
(**a**) The fluorescence images of biofilm after 1, 2, and 3 d (1: control groups; 2: OP-145; 3: vancomycin); (**b**) integrated optical density (IOD) of biofilm after 1, 2, and 3 d (1: control groups; 2: OP-145; 3: vancomycin); (**c**) the percentage of community abundance at the gene level after 3 d (1: control groups; 2: OP-145; 3: vancomycin).

**Figure 5:**
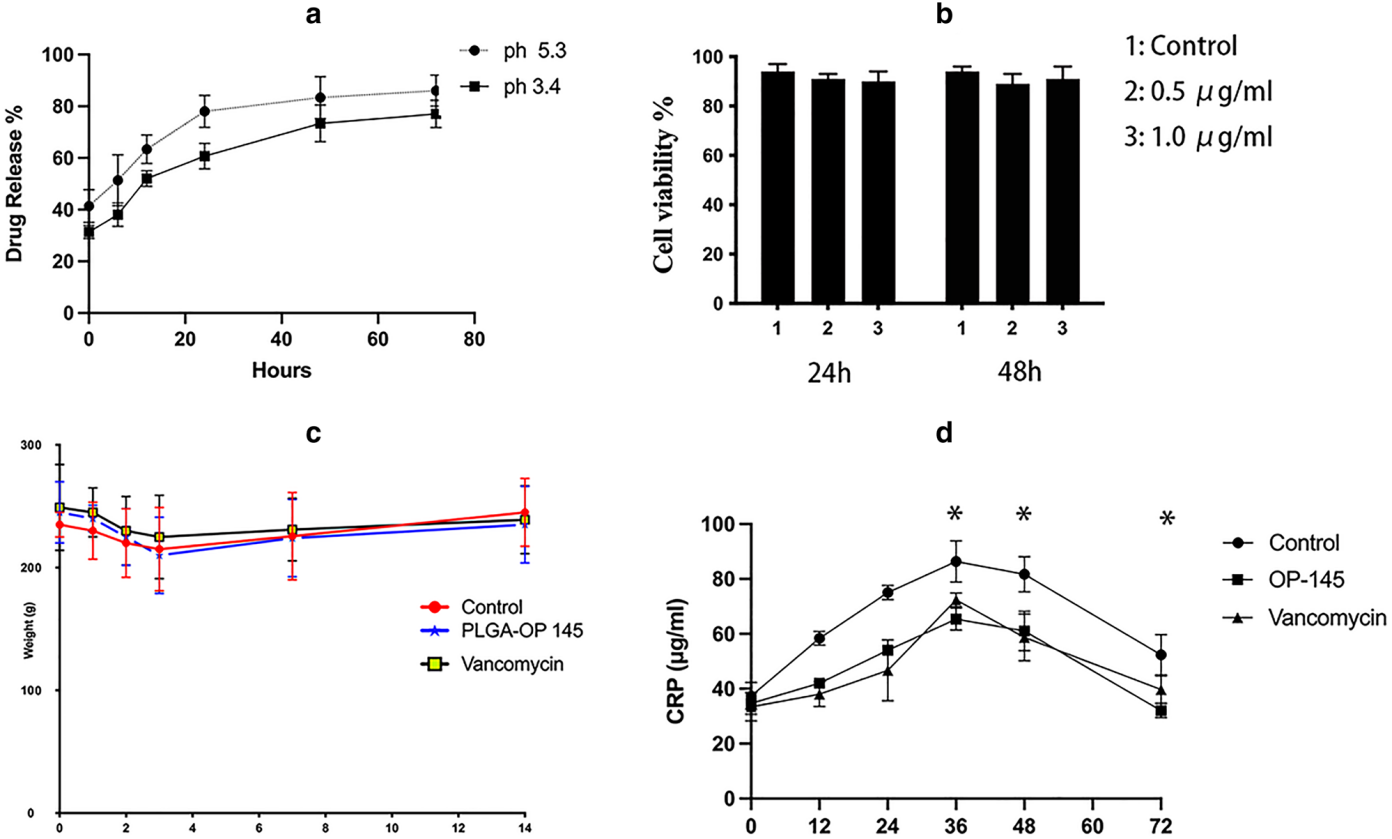
(**a**) The drug release of OP-145 at pH values of 5.3 and 3.4 at 0, 6, 12, 24, 48, and 72 h; (**b**) the cytotoxicity of OP-145 (0.5 and 1 µg/mL) to bone marrow stromal cell; (**c**) the weight of the OP-145, control animals, and vancomycin animals; (**d**) CRP level of OP-145, control animals, and vancomycin animals at 12, 24, 36, 48, and 72 h.

Figure 4 shows the biofilms of the three groups at 1, 2, and 3 d. The fluorescence image showed that OP-145 and vancomycin exerted strong anti-biofilm effects on clinical isolates at 1, 2, and 3 d, which was instant with the IOD values (Fig. 4a-c, *P* < 0.05). The community at the gene level showed that the variety of biofilms was reduced by the OP-145 and vancomycin. In the drug release test, the OP-145 PLGA microspheres could slowly release OP-145 at pH = 3.4 and 5.3 (Fig. 5a). In the MTT test, the 0.5 concentration and 1 µg/mL of OP-145 showed no toxicity on bone marrow stromal cells (BMSCs) (Fig. 5b). The weight of the OP-145, control animals, and vancomycin animals showed no significant differences between groups overall or at any time interval according to repeated measures two-way ANOVA (Fig. 5c). The CRP levels peaked at 36 h, and the OP-145 and vancomycin groups began to exert a lower level of CRP (Fig. 5d, *P* < 0.05).

## Discussion

Infection is one of the most intractable problems in soft tissues, bones, and joints^25^. In addition, infections are associated with increased medical expenses or concurrent severe joint function disorders. The main pathogens in trauma and orthopedic infections are gram-positive cocci (*Staphylococcus aureus* and *Streptococcus pyogenes*)^26^. Wound bacterial biofilm is unique due to its significant resistance to antibiotics and other antibacterial agents^27^. Data show that 65–80% of wound infections are related to bacterial biofilms^28^. Bacterial biofilms have also become one of the main reasons for poor treatment outcomes, delayed healing of infected wounds, surgery, and local administration of antibiotics. At present, the methods to resolve wound bacterial biofilms include local mechanical debridement and destruction, bioengineering replacement therapy, negative pressure therapy, local drugs, etc.^29^. The above methods have certain therapeutic effects; however, bacterial biofilms are still a challenge in the clinical treatment of chronic infections. In our experiments, the OP-145 PLGA microspheres exerted strong anti-biofilm effects on clinical isolated pathogens, the results were consistent with those of previous studies^30,31^.

The main sources of bone infection include endogenous pathogens such as respiratory tract, body surface, urogenital system, and exogenous infections such as wound foreign bodies and air pollution^32^. Clinical reports show that exogenous and nosocomial infection rates can be decreased significantly through air purification^33^. Endogenous infections should be treated with antibiotics. Studies have confirmed that antibiotics in the perioperative period of orthopedic infections can significantly improve the infection, especially in aseptic surgery with large injuries and lengthy and complex surgeries^34^. Antimicrobial peptides (Amps) have been introduced in recent years. Amps is a kind of peptide with great development potential as new antibiotics. It is encoded by genes and synthesized by ribosomes. Antimicrobial peptides are small cationic peptides against infection by external pathogens, produced by the host’s innate immune defense system, and are an important effector molecule of innate immunity^35^. Natural antimicrobial peptides have a broad antibacterial spectrum, especially against clinical isolated multi-drug-resistant bacteria^36^. Moreover, the antibacterial peptide has good thermal stability and water solubility, with no toxic effects on normal cells of animals^37^. OP-145, a synthetic antimicrobial peptide developed from a screen of the human cathelicidin LL-37, exhibits strong antibacterial activity against *Staphylococcus aureus*^38^. As a key factor in preventing bone infection, the application method has gradually changed from previous systemic high-dose medications to the current local medications. At present, local drug use mainly adopts the methods of local implantation of antibiotics and slow-release antibiotics with various carriers^39^. PLGA, as a nano copolymer with excellent biocompatibility and biodegradability, has been approved by FDA for clinical applications^40^. Previous studies have shown that antimicrobial peptide-loaded PLGA could significantly reduce the bacteria biofilm formation. In our present study, the PLGA microspheres containing the antimicrobial peptide OP-145 could slowly release the antimicrobial peptides exerted strong anti-infection effects in vivo, indicating their potential value in controlling bone infections.

In the present study, we only tested the pathogenic bacteria from only one person; however, the etiology of infection of femoral fracture may be more complicated because the bacteria in each infection are not the same. Therefore, the results require further research. Meanwhile, in clinic the chronic infections after fracture may last for 28 days or more, the rats model needs to be improved. In the future, we will also consider all aspects to design experiments in vivo.

## Conclusion

OP-145 PLGA microspheres could slowly release antimicrobial peptides and significantly reduce biofilm formation and levels of inflammatory factors. These findings indicate that OP-145 PLGA microspheres may be one of the promising compounds in the control of femoral fracture infection in vivo.

## Data Availability

The authors confirm that the data supporting the findings of this study are available within the article and the datasets used during the current study are available from the corresponding author on reasonable request.
